# Combined detection of peripheral blood VEGF and inflammation biomarkers to evaluate the clinical response and prognostic prediction of non-operative ESCC

**DOI:** 10.1038/s41598-021-94329-8

**Published:** 2021-07-27

**Authors:** Yuanyuan Ma, Xinyu Su, Xin Li, Xiaohui Zhi, Kan Jiang, Jianhong Xia, Hongliang Li, Chen Yan, Liqing Zhou

**Affiliations:** Department of Radiation Oncology, The Affiliated Huai’an Hospital of Xuzhou Medical University, The Second People’s Hospital of Huai’an, Huai’an, China

**Keywords:** Cancer models, Gastrointestinal cancer, Tumour angiogenesis, Tumour biomarkers, Biomarkers

## Abstract

An association between angiogenesis/inflammation status and tumor has been reported in various types of cancer. This study sought to assess the role of peripheral blood VEGF and some inflammation biomarkers in evaluating clinical response and prognosis in patients with non-operative esophageal squamous cell carcinoma (ESCC). Peripheral blood of 143 patients with non-operative ESCC at our institute was dynamically collected at 5 time points including 1 day before radiotherapy, during radiotherapy (15f), at the end of radiotherapy, 1 month after radiotherapy, and 3 months after radiotherapy. VEGF expression in the peripheral blood was detected and related inflammation biomarkers such as GPS, CAR and CLR were counted. Logistic regression and Cox regression were implemented respectively to analyze the correlation of each predictor with clinical response and prognosis. The performance of combined testing was estimated using AUCs. Based on independent predictors, a nomogram prediction model was established to predict the probabilities of 1- and 2-year PFS of patients. The effectiveness of the nomogram model was characterized by C-index, AUC, calibration curves and DCA. VEGF and CLR levels at the end of radiotherapy were independent predictors of clinical response, while VEGF and GPS levels at 3 months after radiotherapy were independent prognostic predictors. The efficacy of combined detection of VEGF and CLR is superior to the single detection in evaluating clinical response and prognosis. The nomogram showed excellent accuracy in predicting PFS. The combined detection of VEGF and CLR at the end of radiotherapy can be used to evaluate the clinical response of patients with non-operative ESCC, and the combined detection of VEGF and GPS 3 months after radiotherapy can be used to predict the prognosis. Implemented by nomogram model, it is expected to provide practical and reliable method to evaluate the clinical response and prognosis of patients with non-operative ESCC tool.

## Introduction

Esophageal cancer is one of the most common malignant tumors in the digestive system, with the seventh and sixth morbidity rates in the world^1^. Adenocarcinoma and squamous cell carcinoma are the two main important pathological types of esophageal cancer, and about 90% of Chinese patients are esophageal squamous cell carcinomas (ESCC). Radiotherapy (RT) and chemotherapy are the main treatment methods for patients with local advanced stage or patients who cannot be resected or refuse surgery^2^. However, currently, there are no established criteria for the clinical response evaluation and prognosis of the response to radiotherapy and chemotherapy for non-operative esophageal cancer. As the molecular biology of esophageal cancer was further explored^3,4^, researchers found that certain molecules related to angiogenesis and inflammation might contribute to cancer recurrence and metastasis. Therefore, finding a specific and sensitive biomarkers and method to evaluate the clinical response and prognosis of non-operative ESCC patients becomes imperative.

Sustained angiogenesis and tumor promotion inflammation are two significant hallmarks of cancer^5^, working together to coordinate the oncogenesis and development of tumor. VEGF (Vascular Endothelial Growth Factor), a mitogen activator secreted by vascular endothelial cells, plays an important role in the formation of highly permeable, immature and poorly perfused tumor-related blood vessels, which is closely related to the recurrence and metastasis of cancer^6^. In previous studies on esophageal cancer^7–9^, sufficient research has confirmed that overexpression of VEGF is significantly correlated with tumor stage, invasion depth, lymph node status and metastasis of the tumor. In recent years, the effect of tumor promotion inflammation has been widely recognized on solid tumors. The inflammation biomarkers based on CRP (C Reactive Protein) or ALB (Albumin) or lymphocyte level, such as GPS (Glasgow Prognostic Score), CAR (C Reactive Protein to Albumin Ratio) and CLR (C Reactive Protein to Lymphocyte Ratio) etc. are closely related to the prognosis in a variety of cancers including esophageal cancer^10–13^. They can reflect the inflammation, nutritional and immune status of patients. The sensitivity and specificity for clinical response evaluation and prognostic prediction of most inflammation biomarkers are only 50–70%^11,13,14^, with poor clinical application value. Considering the interconnection between sustained angiogenesis and tumor promotion inflammation is explained by the secretion of VEGF^15^ and inflammatory cytokine such as leukocytes and CRP that in turn amplify tumorigenic signal via CD64/PI3k/Akt and MAPK/ERK signaling pathways^16^, we analysis them together by combined detection.

In our study, we combined detection peripheral blood VEGF and inflammation biomarkers to the clinical response assessment and prognosis prediction of patients with non-operative ESCC. The aim of our study was to evaluate the clinical response and prognosis of non-operative ESCC patients by monitoring simple biomarkers, and to establish a nomogram prediction model for prognostic prediction. It is useful for the classification and management of patients and illustrative for early treatment strategy.

## Methods

### Patient selection

**Figure 1:**
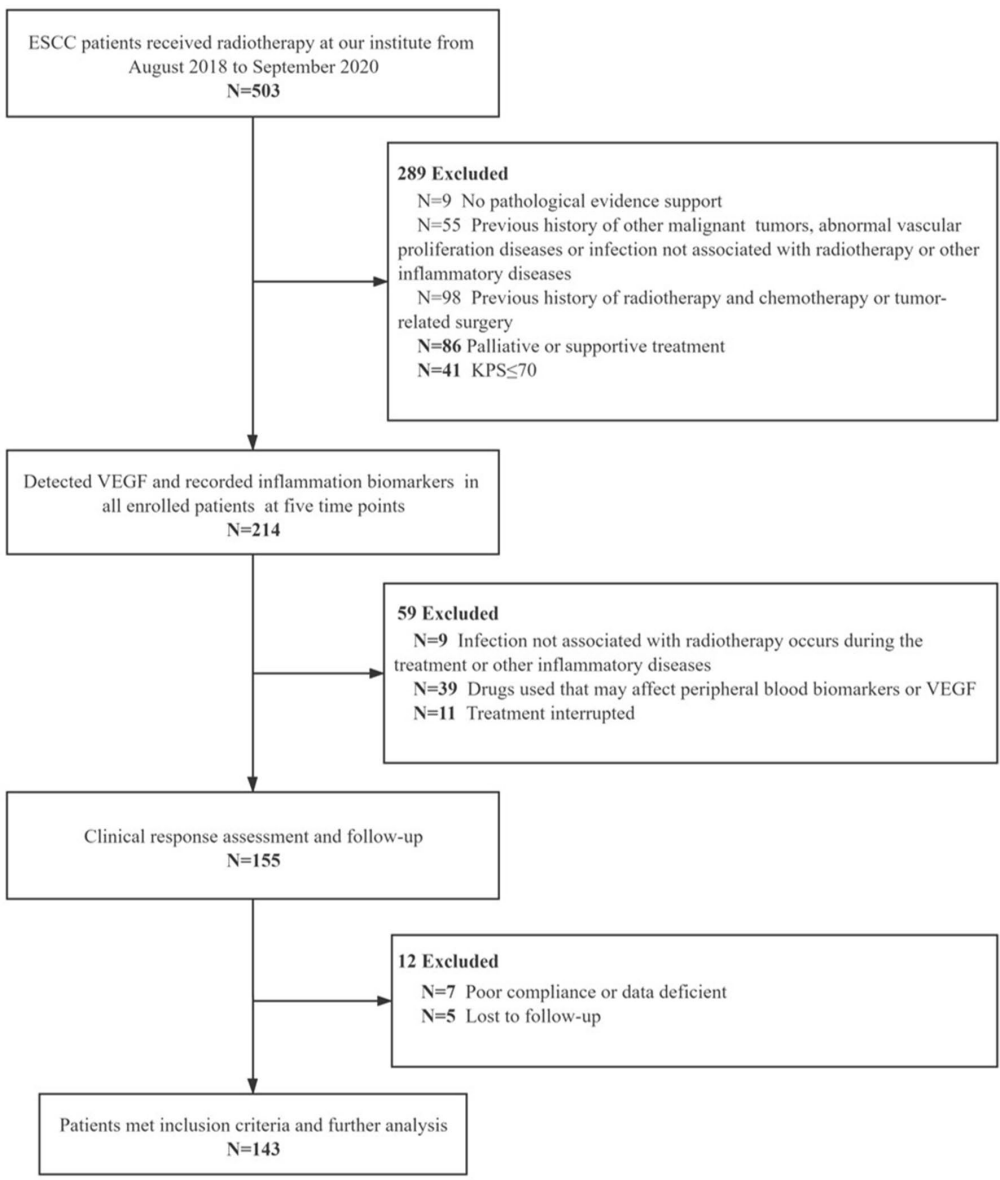
Patient enrollment flow chart.

A total of 503 ESCC patients received radiotherapy at our institute from August 2018 to September 2020, and classified by the 8th edition AJCC/UICC classification^17^. Exclusion criteria for patients based on regular follow-up were as following: (1) no pathological evidence support; (2) previous history of other malignant tumors, abnormal vascular proliferation diseases (such as asthma, retinopathy, liver disease, pleural effusion, peripheral vascular diseases etc.) or infection not associated with radiotherapy or other inflammatory diseases (such as pneumonia, ulcerative colitis, connective tissue diseases, rheumatism, acute infections etc.); (3) previous history of radiotherapy and chemotherapy or tumor-related surgery; (4) palliative or supportive treatment; (5) KPS (Karnofsky) ≤ 70 points; (6) infection not associated with radiotherapy occurs during the treatment or other inflammatory diseases; (7) drugs used that may affect peripheral blood biomarkers or VEGF (such as recombinant human granulocyte stimulating factor, thrombopoietin, anti-angiogenic drugs etc. used within 1 week before blood cell samples collection); (8) poor compliance or data deficient; (9) lost to follow-up. Through the above filters, a total of 143 patients were included in this study. Peripheral blood samples were collected as planned. Blood routine, blood biochemical and imaging examinations were regularly conducted. The flow chart of patient enrollment is shown in Fig. 1.

### VEGF dectection

4 ml of peripheral blood from all enrolled patients was collected at five time points including 1 day before radiotherapy, during radiotherapy (15f), the end of radiotherapy, 1 month after radiotherapy, and 3 months after radiotherapy. Peripheral blood samples were placed in the anticoagulant EDTA tube for 30 min, centrifuged at 3500 r/min for 10 min, and stored in a refrigerator at minus 80 °C. VEGF detection kits were provided by Beijing Jianping Jinxing Biotechnology Co., Ltd. (product registration number: Jingxi Zhuzhun 20152400398) and stored at 2–8 °C. Samples were tested by enzyme-linked immunosorbent assay (ELISA method). The brief operation steps are: (1) preparation of lotion; (2) dilution of calibrator; (3) adding sample and incubating wash plate; (4) adding enzyme; (5) washing plate; (6) color development; (7) termination; (8) calibration and measurement (450 nm wavelength of microplate reader, reference wavelength 630 nm); (9) calculating and recording VEGF value according to the calibration curve and the OD (Absorbance) value measured by the microplate reader.

### Data collection and definition

The age, gender, differentiation, imaging and other clinical data of all patients as well as the expression values of related inflammation indicators were found and recorded through the Website V1.1 of The Hospital (Nanjing Yijiantong Information Technology Co., LTD.) and subsequent follow-up. The relevant inflammation biomarkers were defined as follows:

- *GPS* The value was 1 for elevated C reactive protein combined with low albumin; only one abnormal phenomenon got 1. Both normal indicators were assigned with 0.
- *CAR* The ratio of C reactive protein to albumin.
- *CLR* The ratio of C reactive protein to lymphocyte.

### Treatment details

The radiotherapy was delivered with 6 MV photon beams for IMRT (Intensity Modulated Radiotherapy) in Eclipse treatment planning system (Varian Medical Systems, AAA 11.0). All patients underwent a baseline enhanced CT (Siemens Medical Systems, Iselin, NJ) scan before the treatment in the supine position. GTV (Gross Tumor Volume) obtained the primary lesion and positive regional lymph nodes (included parattracheal, posterior, anterior mediastinal, subcarina, paraesophagus, pericardium, subpulmonary ligament, and recurrent laryngeal nerve lymph nodes). CTV (Clinical Tumor Volume) was expanded by a 0.5 cm radial margin around GTV, and was extended by 3.0–5.0 cm in the proximal and distal direction. PTV (Planning Tumor Volume) provided a 0.3–0.5 cm margin around CTV. The vital organs, including the spinal cord, heart, and bilateral lungs, were mapped and the optimal treatment plan was determined by experienced clinicians and physiotherapists according to the dose-volume histogram (DVH) and isodose curve. The prescription dose of PTV was 60–64 Gy, 1.8–2.2 Gy/day, 5 days/week. Considering the patient’s age, basic state and other conditions, appropriate individualized treatment was conducted. The target area and dose can be reduced if necessary. The chemotherapy regimen uses the "TP" regimen, namely: liposomal paclitaxel 45–60 mg/m^2^, on the first day; cisplatin 20–25 mg/m^2^, from the first to third day, ivgtt (intravenously guttae), 21 days a cycle. Two cycles of chemotherapy were started simultaneously on the first day of radiotherapy until the end of radiotherapy.

### Clinical response assessment

Response evaluation criteria in solid tumors (RECIST 1.1) was adopted^18^. The clinical response assessment was evaluated 2–3 weeks after the end of radiotherapy. Compared with the longest diameter of the primary lesion measured by enhanced CT scan in sagittal image before radiotherapy, the clinical response was divided into complete response (CR), partial response (PR), stable disease (SD) and progressive disease (PD). CR showed that all lesions disappeared. PR indicated that the total maximum diameter of target lesions was reduced by 30%, PD revealed that the total maximum diameter of target lesions increased by 20% or new lesions appeared. SD showed that the reduction of target lesions did not reach PR or the increase failed reaching PD. In addition, the smoothness of the mucous membrane and the surrounding tissues respectively were evaluated combining the X-ray barium meal and MRI (Magnetic Resonance Imaging) evaluation of patients to increase the accuracy and avoid omissions.

### Follow-up

The adverse events were evaluated according to the National Cancer Institute Common Terminology Criteria for Adverse Events (version 4.0). According to the 8th edition AJCC/UICC^17^, N+ is defined as a lymph node with a short diameter of > 8 mm identified on enhanced CT scan before radiotherapy. At the end of radiotherapy, an enhanced CT scan was performed again to measure the short diameter of the positive lymph nodes namely the short diameter of residual lymph node (SDRLN). Telephone follow-up, outpatient follow-up etc. were used for follow-up. Follow-up were arranged a month after radiotherapy, then every 3 months in the first year, and every 6 months from the second year until the end of the follow-up or the end of the study. The time of recurrence, metastasis, or death of the patient was recorded. The deadline for follow-up was September 1, 2020. The PFS (progression-free survival) and OS (overall survival) were calculated.

### Statistical analysis

Descriptive statistics of patient baseline clinicopathological characteristics are expressed in medians and 95% confidence intervals (CIs). Survival analysis uses a log-rank test. The ROC (Receiver operating) curve was used to compare the area under the curve (AUC) at different time nodes. For each variable, the optimum cutoff value corresponding to the time node with the maximum AUC value was calculated using X tile 3.6.1 (Yale University, New Haven, CT, USA). Univariate and multivariate logistic regression model analysis was implemented to analyze the correlation between the risk factors and clinical response. The risk factors for PFS were calculated by univariate and multivariate cox regression model. To compare the accuracy of clinical response prediction and PFS between combined markers and independent markers, the ROC curve was applied. SPSS 24.0 (SPSS Inc., Chicago, IL, USA) was used to analyze the data. All tests were two-sided tests, p < 0.05 was considered statistically significant.

Based on the Cox regression model analysis, a nomogram prediction model was developed by using the R × 64 3.6.3 (R Foundation for Statistical Computing, Vienna, Austria). It was further evaluated through calculating the value of C-index (concordance index), ploting ROC curve, calibrating curve and and conducting DCA (decision curve analysis). The installation packages involved are: Hmisc, survival, rms, pROC, lattice, Formula, ggplot2 and rmda.

### Ethics approval and consent to participate

Informed consent was obtained from all enrolled patients. Our study was conducted in accordance with the Declaration of Helsinki, and was approved by the Ethics Committee of the Affiliated Huai'an Hospital of Xuzhou Medical University.

## Results

### Patient characteristics

The clinicopathological baseline characteristics of patients included in this study before treatment and their relationship with PFS were shown in Table 1. The median age was 73. Overall, 69 (48.3%) patients were older than 73 years old. 86 (60.1%) patients were male, and 57 (39.9%) were female. Among the entire patient cohort, 83 patients (58.0%) underwent radiotherapy (RT), and 60 patients (42.0%) underwent chemoradiotherapy (CRT). Whereas undifferentiated (median, 6.7; 95% CI 4.5–8.9), SDRLN > 0.4 cm (median, 6.7; 95% CI 4.5–8.9) and TNM stage III-IV (median, 5.5; 95% CI 3.5–7.5) were associated with poor PFS (p < 0.0001). Moreover, the median follow-up for OS and PFS was 12.6 months (range 3.5–31.2) and 7.8 months (range 1.5–30.6) respectively.

**Table 1:** Baseline clinicopathological characteristics of patients with ESCC and log-rank test.

Characteristics	Level	Number (%)	PFS (months) mean (95% CI)	p value
Age (years)				0.845
	> 73 (median)	69 (48.3)	13.8 (10.9–16.7)	
	≤ 73	74 (51.7)	14.6 (7.0–22.2)	
Sex				0.407
	Male	86 (60.1)	14.3 (8.6–20.0)	
	Female	57 (39.9)	14.2 (10.7–17.7)	
Treatment				0.816
	RT	83 (58.0)	14.3 (8.7–19.9)	
	CRT	60 (42.0)	11.9 (6.6–17.2)	
Differentiation				**< 0.0001**
	Differentiated	98 (68.5)	21.7 (16.3–27.1)	
	Undifferentiated	45 (31.5)	6.7 (4.5–8.9)	
Tumor length (cm)				0.318
	> 5 (median)	48 (33.6)	10.4 (5.6–15.2)	
	≤ 5	95 (66.4)	14.5 (8.4–20.6)	
Tumor location				0.138
	Cervical	10 (7.0)	13.1 (7.2–20.3)	
	Upper thoracic	36 (25.2)	11.9 (4.2–19.6)	
	Middle thoracic	43 (30.1)	11.9 (4.2–19.6)	
	Lower thoracic	54 (37.8)	11.3 (5.7–16.9)	
SDRLN (cm)				**< 0.0001**
	> 0.4 (median)	57 (39.9)	6.7 (4.5–8.9)	
	≤ 0.4	86 (60.1)	10.7 (10.3–13.2)	
TNM				**< 0.0001**
	I–II	89 (62.2)	26.8 (19.6–34.0)	
	III–IV	54 (37.8)	5.5 (3.5–7.5)	
Adverse events (> Grade 2)				0.091
	Yes	33 (23.1)	7.8 (7.0–8.6)	
	No	110 (76.9)	14.5 (8.9–20.1)	

*RT* radiotherapy, *CRT* chemoradiotherapy, *SDRLN* short diameter of residual lymph node.

The bold entries represent statistically significant.

### Optimal cutoff values of VEGF and the inflammation biomarkers

**Figure 2:**
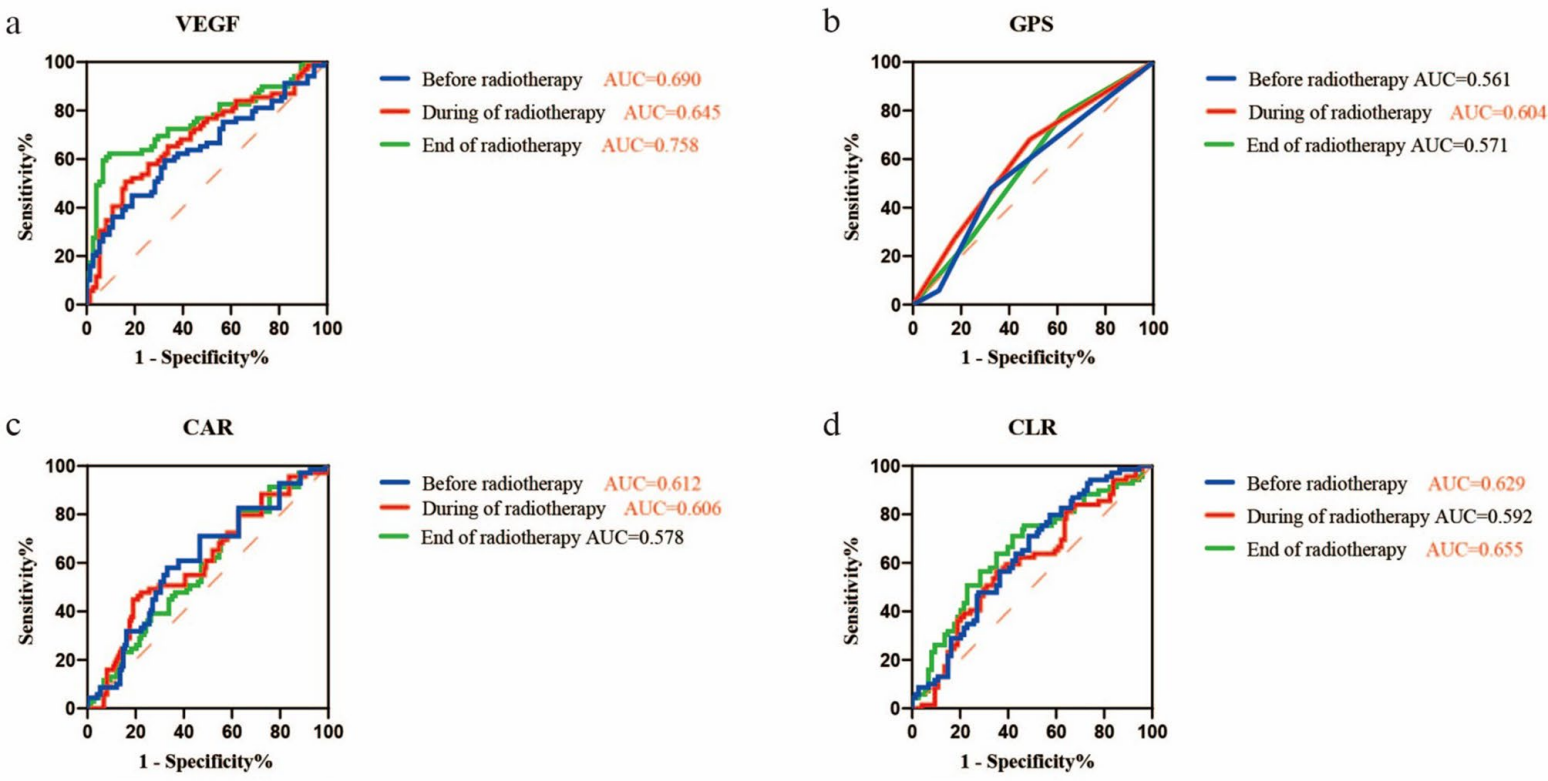
The predictive ability of VEGF and the inflammatory biomarkers were compared by ROC curves. (**a**) The AUCs of VEGF before, during and end of radiotherapy were 0.690 (p < 0.0001), 0.645 (p = 0.003), 0.758 (p < 0.0001) respectively. (**b**) The AUCs of GPS before, during and the end of radiotherapy were 0.561 (p = 0.212), 0.604 (p = 0.031), 0.571 (p = 0.144) respectively. (**c**) The AUCs of CAR before, during and the end of radiotherapy were 0.612 (p = 0.021), 0.606 (p = 0.029), 0.578 (p = 0.106) respectively. (**d**) The AUCs of CLR before, during and the end of radiotherapy were 0.629 (p = 0.008), 0.592 (p = 0.057), 0.655 (p = 0.001) respectively. The red entries represent statistically significant.

**Figure 3:**
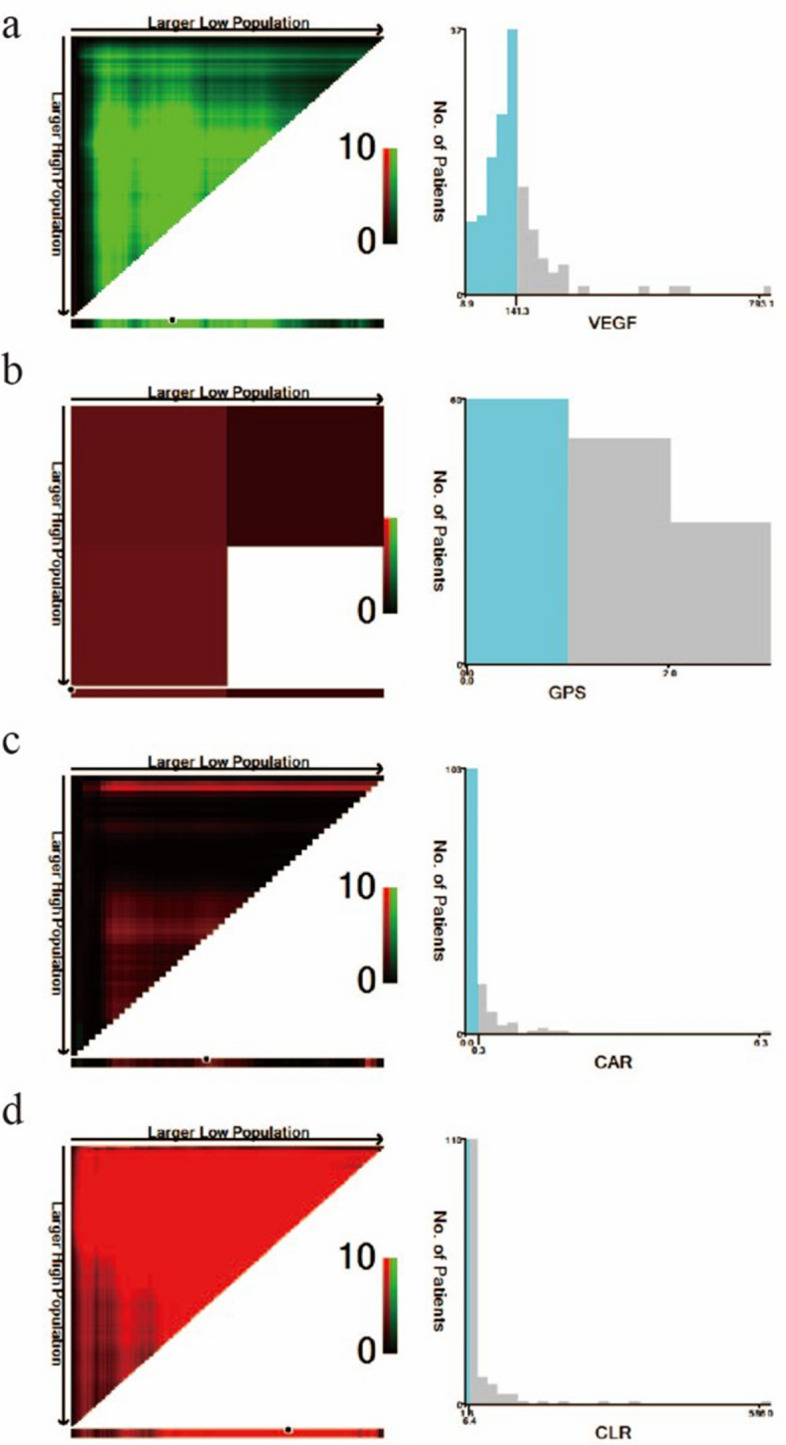
4 X-tile analysis of PFS was performed by the X-tile program to determine the optimal cutoff values for VEGF, GPS, CAR, and CLR. The sample of ESCC patients was equally divided into training and validation sets. X-tile plots of training sets are shown in the left panels, with plots of matched validation sets shown in the small inset. The optimal cut-off values highlighted by the black circles in the left panels are shown in histograms of the entire cohort (right panels). p values were determined by using the cutoff values defined in training sets and applying them to validation sets. The optimal cutoff values for VEGF, GPS, CAR, and CLR were 141.3, 0, 0.3, and 6.4, respectively. (**a**) VEGF, (**b**) GPS, (**c**) CAR, and (**d**) CLR.

As shown in Fig. 2, the ROC curves were generated to compare the AUC values of each biomarker during radiotherapy, thus to find the best time point for diagnostic performance. Although almost all time points of the biomarkers could predict poor PFS, each biomarker has different diagnostic performance at various time points. The AUC value of VEGF after radiotherapy (AUC = 0.758, p < 0.0001) was higher than that before radiotherapy (AUC = 0.690, p < 0.0001) and during radiotherapy (AUC = 0.645, p = 0.003). The ROC of GPS before and end of radiotherapy did not differ significantly. GPS during radiotherapy (AUC = 0.604, p = 0.031) was still statistically significant in predicting prognosis. The AUC value of CAR before radiotherapy (AUC = 0.612, p = 0.021) was higher than that during radiotherapy (AUC = 0.606, p = 0.029). The AUC value of CLR end of radiotherapy (AUC = 0.655, p = 0.001) was higher than that before radiotherapy (AUC = 0.629, p = 0.008). Based on the aforementioned findings, we chose the time node with the maximum AUC value of each biomarker to calculate the optimum cutoff value as shown in Fig. 3. The optimum cutoff value of VEGF, GPS, CAR and CLR was 141.3, 0, 0.3, 6.4 respectively. We then grouped them into high-, and low-value group.

### Association of VEGF and the inflammation biomarkers with clinical response

**Figure 4:**
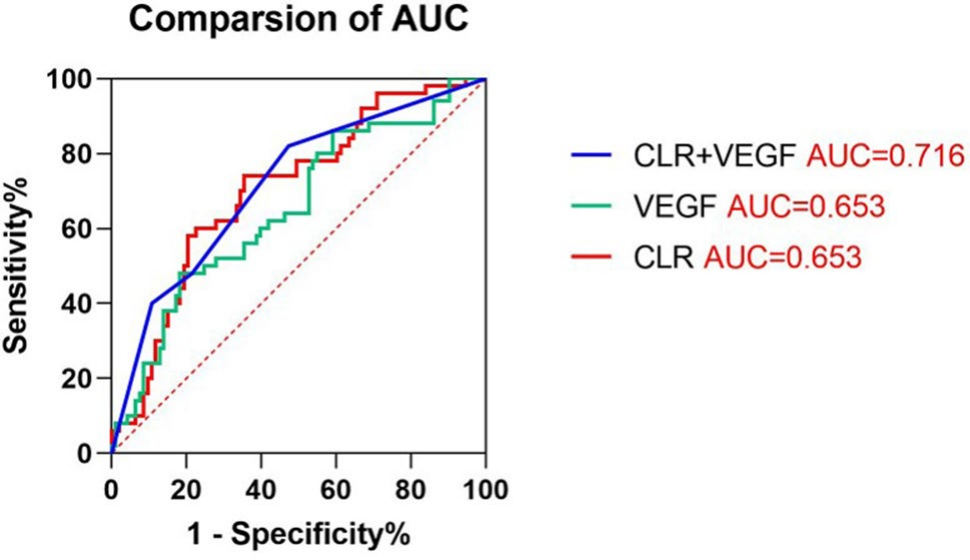
Comparison of the AUCs for the clinical response assessment of combined detection of VEGF and CLR (AUC = 0.716, p < 0.0001), VEGF (AUC = 0.653, p = 0.003), CLR (AUC = 0.703, p < 0.0001) at the end of radiotherapy for ESCC patients. The red entries represent statistically significant.

The results of univariate analysis and multivariate logistic regression model analysis were presented in Table 2. Statistically significant variables in univariate analysis were included in multivariate analysis. The differentiated (HR = 0.193; 95% CI 0.071–0.528; p = 0.001), SDRLN > 0.4 cm (HR = 3.511; 95% CI 1.323–9.319; p = 0.012), TNM stage I–II (HR = 0.010; 95% CI 0.002–0.045; p < 0.0001), high expression of VEGF end of radiotherapy (HR = 2.814; 95% CI 1.040–7.615; p = 0.042) and CLR end of radiotherapy (HR = 3.126; 95% CI 1.164–8.393; p = 0.024) were related to poor clinical response. Further comparison of the ROC curves (Fig. 4) showed that the AUCs for clinical response were 0.716 (p < 0.0001), 0.653 (p = 0.003), 0.703 (p < 0.0001) for combined detection of VEGF end of radiotherapy and CLR end of radiotherapy, VEGF end of radiotherapy, CLR end of radiotherapy, respectively. The combined marker had a specificity of 52.7% (CLR end of radiotherapy: 64.5%, VEGF end of radiotherapy: 81.7%) and a sensitivity of 82.0% (CLR end of radiotherapy: 74.0%, VEGF end of radiotherapy: 48.0%). Moreover, the poor application value of combined detection of VEGF and CAR for the clinical response assessment was shown in Supplementary Fig. S1.

**Table 2:** Univariate and multivariate logistic regression analysis of clinical response.

Variables	Univariate			Multivariate		
	OR	95% CI	p value	OR	95% CI	p value
**Age (years)**			0.908			
> 73 (median)	1.038	0.550–1.959				
≤ 73	1					
**Sex**			0.597			
Male	1.192	0.621–2.286				
Female	1					
**Treatment**			0.490			
RT	0.797	0.419–1.516				
CRT	1					
**Differentiation**			**< 0.0001**			**0.001**
Differentiated	0.139	0.067–0.291		0.193	0.071–0.528	
Undifferentiated	1			1		
**Tumor length (cm)**			**< 0.0001**			0.554
> 5 (median)	3.702	1.855–7.390		0.764	0.314–1.861	
≤ 5	1			1		
**Tumor location**						
Cervical	0.650	0.171–2.467	0.527			
Upper thoracic	0.850	0.377–1.919	0.696			
Middle thoracic	0.765	0.351–1.664	0.499			
Lower thoracic	1					
**SDRLN (cm)**			**< 0.0001**			**0.012**
> 0.4 (median)	7.780	3.712–16.310		3.511	1.323–9.319	
≤ 0.4	1			1		
**TNM stages**			**< 0.0001**			**< 0.0001**
I–II	0.006	0.001–0.022		0.010	0.002–0.045	
III–IV	1			1		
**Adverse events (> Grade 2)**			**0.033**			0.920
Yes	2.248	1.069–4.726		1.051	0.399–2.772	
No	1			1		
**Before radiotherapy**						
VEGF (high/low group)	2.159	1.096–4.253	**0.026**	1.718	0.711–4.147	0.229
GPS (high/low group)	3.093	1.576–6.069	**0.001**	1.375	0.421–4.487	0.598
CAR (high/low group)	3.101	1.475–6.517	**0.003**	0.806	0.107–6.050	0.834
CLR (high/low group)	2.787	1.366–5.686	**0.005**	2.094	0.320–13.717	0.441
**During radiotherapy**						
VEGF (high/low group)	1.330	0.700–2.527	0.383			
GPS (high/low group)	3.014	1.508–6.023	**0.002**	1.400	0.474–4.132	0.542
CAR (high/low group)	2.872	1.461–5.648	**0.002**	1.150	0.391–3.376	0.800
CLR (high/low group)	2.456	1.098–5.533	**0.029**	1.192	0.398–3.568	0.753
**End of radiotherapy**						
VEGF (high/low group)	0.266	0.132–0.536	**< 0.0001**	2.814	1.040–7.615	**0.042**
GPS (high/low group)	2.408	1.153–5.031	**0.019**	1.475	0.501–4.342	0.481
CAR (high/low group)	2.462	1.269–4.776	**0.008**	0.985	0.335–2.891	0.977
CLR (high/low group)	5.530	2.694–11.350	**< 0.0001**	3.126	1.164–8.393	**0.024**

*RT* radiotherapy, *CRT* chemoradiotherapy, *SDRLN* short diameter of residual lymph node, *VEGF* vascular endothelial growth factor, *GPS* Glasgow prognostic score, *CAR* C reactive protein/albumin ratio, *CLR* C reactive protein/lymphocyte ratio, *OR* odd ratio, *CI* confidence interval.

The bold entries represent statistically significant.

### Prognostic value of VEGF and the inflammation biomarkers

**Figure 5:**
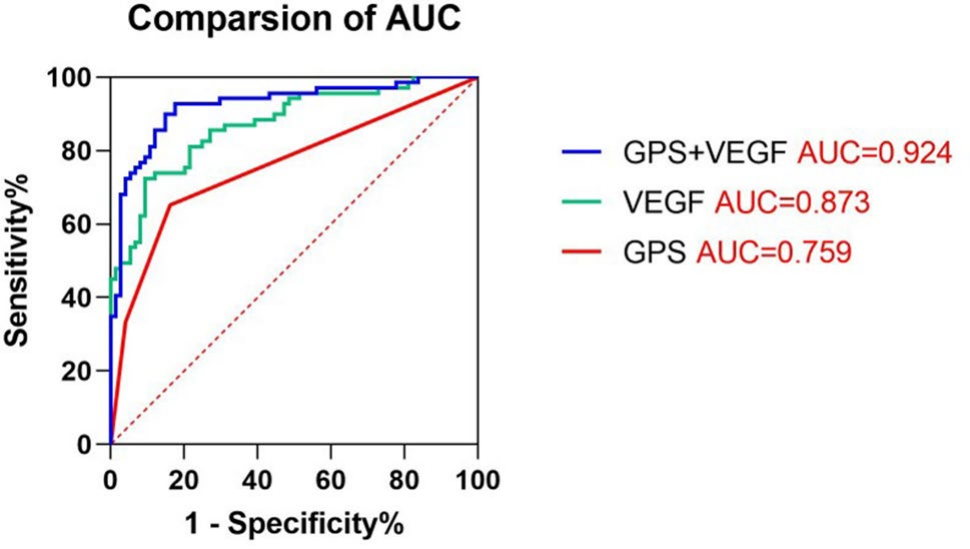
Comparison of the AUCs for prognostic prediction of combined detection of VEGF and GPS (AUC = 0.924, p < 0.0001), VEGF (AUC = 0.873, p < 0.0001), GPS (AUC = 0.759, p < 0.0001) 3 months after radiotherapy for ESCC patients. The red entries represent statistically significant.

**Figure 6:**
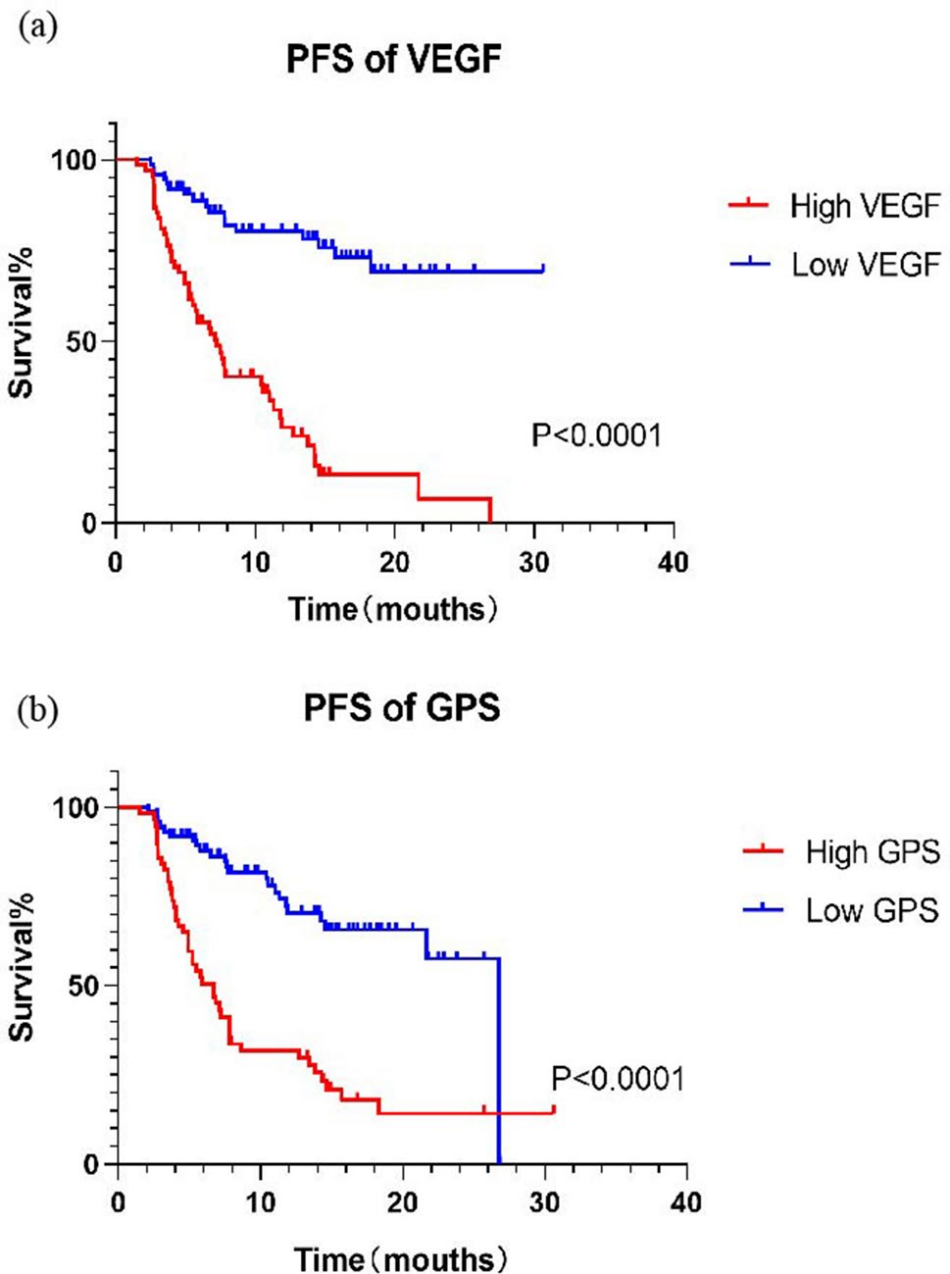
Kaplan–Meier analysis on different VEGF/GPS groups (high and low) of all enrolled patients. (**a**) The high VEGF group was significantly associated compared to low VEGF group with poor prognosis (p < 0.0001) of patients. (**b**) The high GPS group was significantly associated compared to low GPS group with poor prognosis (p < 0.0001) of patients.

According to the univariate and multivariate Cox regression analysis results of the PFS from ESCC patients, this significant variables were related to poor PFS (Table 3), including differentiated (HR = 0.493; 95% CI 0.292–0.831; p = 0.008), SDRLN > 0.4 cm (HR = 3.076; 95% CI 1.718–5.507; p < 0.0001), TNM stage I–II (HR = 0.421; 95% CI 0.230–0.770; p = 0.005), high expression of VEGF before radiotherapy (HR = 1.819; 95% CI 1.040–3.182; p = 0.036), VEGF end of radiotherapy (HR = 2.174; 95% CI 1.620–4.803; p < 0.0001), VEGF 1 month after radiotherapy (HR = 4.934; 95% CI 2.600–9.365; p < 0.0001), VEGF 3 months after radiotherapy (HR = 4.095; 95% CI 2.265–7.403; p < 0.0001) and GPS 3 months after radiotherapy (HR = 2.404; 95% CI 1.394–4.146; p = 0.002). Further comparison the ROC curves (Fig. 5) displayed that the AUCs for PFS were 0.924 (p < 0.0001), 0.873 (p < 0.0001), 0.759 (p < 0.0001) for combined detection of VEGF 3 months after radiotherapy and CLR 3 months after radiotherapy, VEGF 3 months after radiotherapy single detection, CLR 3 months after radiotherapy single detection, respectively. The combined marker had a specificity of 82.43% (GPS 3 months after radiotherapy: 83.8%, VEGF 3 months after radiotherapy: 90.5%) and a sensitivity of 92.75% (GPS 3 months after radiotherapy: 65.2%, VEGF 3 months after radiotherapy: 72.5%). A time-to-event analysis showed that patients with a high VEGF/GPS (Fig. 6a,b) were significantly correlated with poor prognosis compared to those with a low VEGF/GPS (Fig. 6a,b) in terms of the PFS. Moreover, the poor application value of combined detection of VEGF and CAR for prognostic prediction was shown in Supplementary Fig. S2.

**Table 3:** Univariate and multivariate Cox regression analysis of PFS.

Variables	Univariate			Multivariate		
	OR	95% CI	p value	OR	95% CI	p value
**Age (years)**			0.846			
> 73 (median)	1.048	0.651–1.687				
≤ 73	1					
**Sex**			0.410			
Male	0.818	0.506–1.320				
Female	1					
**Treatment**			0.816			
RT	0.945	0.587–1.522				
CRT	1					
**Differentiation**			**< 0.0001**			**0.008**
Differentiated	0.336	0.207–0.545		0.493	0.292–0.831	
Undifferentiated	1			1		
**Tumor length (cm)**			0.322			
> 5 (median)	1.280	0.786–2.084				
≤ 5	1					
**Tumor location**						
Cervical	0.253	0.060–1.066	0.061			
Upper thoracic	1.032	0.575–1.852	0.917			
Middle thoracic	0.699	0.390–1.253	0.229			
Lower thoracic	1					
**SDRLN (cm)**			**< 0.0001**			**< 0.0001**
> 0.4 (median)	4.944	2.962–8.253		3.076	1.718–5.507	
≤ 0.4	1			1		
**TNM stage**			**< 0.0001**			**0.005**
I–II	0.189	0.114–0.313		0.421	0.230–0.770	
III–IV	1			1		
**Adverse events (> Grade 2)**			0.095			
Yes	1.589	0.923–2.737				
No	1					
**Before radiotherapy**						
VEGF (high/low group)	2.436	1.411–4.207	**0.001**	1.819	1.040–3.182	**0.036**
GPS (high/low group)	1.616	1.006–2.595	**0.047**	1.218	0.613–2.418	0.547
CAR (high/low group)	1.765	1.060–2.937	**0.029**	1.173	0.560–2.454	0.672
CLR (high/low group)	1.400	0.847–2.312	0.189			
**During radiotherapy**						
VEGF (high/low group)	1.793	1.089–2.951	**0.022**	1.531	0.907–2.584	0.111
GPS (high/low group)	1.725	1.037–2.868	**0.036**	0.675	0.320–1.422	0.301
CAR (high/low group)	1.952	1.215–3.139	**0.006**	1.574	0.822–3.015	0.171
CLR (high/low group)	1.611	0.844–3.073	0.148			
**End of radiotherapy**						
VEGF (high/low group)	4.820	2.934–7.919	**< 0.0001**	2.174	1.620–4.803	**< 0.0001**
GPS (high/low group)	1.956	1.101–3.474	**0.022**	0.501	0.866–2.794	0.140
CAR (high/low group)	1.481	0.914–2.399	0.110			
CLR (high/low group)	2.221	1.358–3.633	0.001	0.423	0.802–2.256	0.261
**1 m after radiotherapy**						
VEGF (high/low group)	7.539	4.215–13.48	**< 0.0001**	4.934	2.600–9.365	**< 0.0001**
GPS (high/low group)	2.094	1.281–3.423	**0.003**	1.155	0.563–2.369	0.695
CAR (high/low group)	2.045	1.268–3.298	**0.003**	1.187	0.612–2.301	0.612
CLR (high/low group)	1.278	0.633–2.582	0.494			
**3 m after radiotherapy**						
VEGF (high/low group)	5.786	3.280–10.20	**< 0.0001**	4.095	2.265–7.403	**< 0.0001**
GPS (high/low group)	3.924	2.384–6.461	**< 0.0001**	2.404	1.394–4.146	**0.002**
CAR (high/low group)	2.951	1.821–4.780	**< 0.0001**	0.706	0.358–1.391	0.314
CLR (high/low group)	3.781	2.277–6.276	**< 0.0001**	1.628	0.757–3.502	0.212

*RT* radiotherapy, *CRT* chemoradiotherapy, *SDRLN* short diameter of residual lymph node, *VEGF* vascular endothelial growth factor, *GPS* Glasgow prognostic score, *CAR* C reactive protein/albumin ratio, *CLR* C reactive protein/lymphocyte ratio, *OR* odd ratio, *CI* confidence interval.

The bold entries represent statistically significant.

### Establishment and evaluation of the nomogram

**Figure 7:**
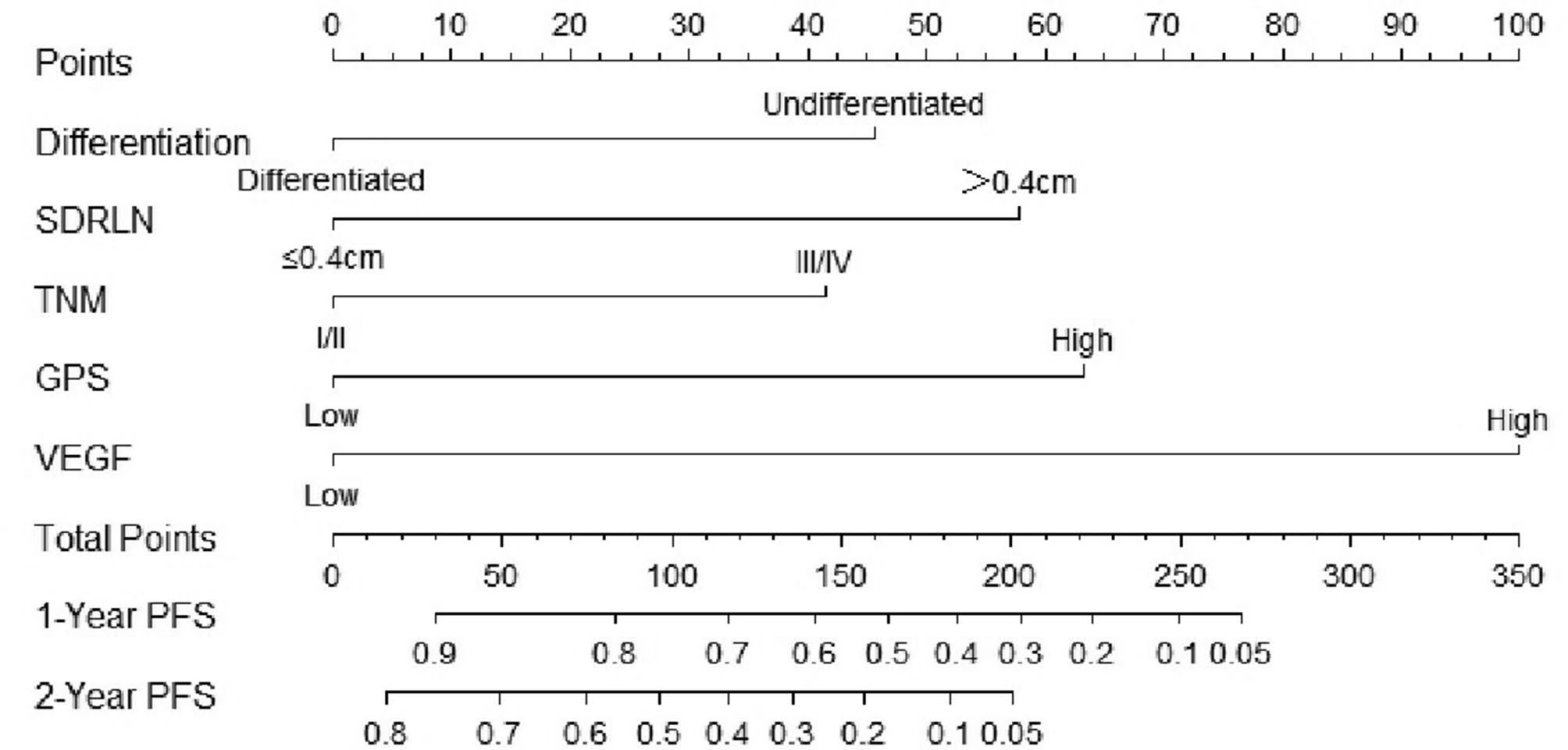
Nomogram predicting the PFS for ESCC patients. For every patient, six lines are drawn upward to determine the points received from the six predictors in the nomogram. The sum of these points is located on the ’Total Points’ axis. In addition, a line is drawn downward to determine the possibility of 1-, and 2-year PFS. Furthermore, according to the total scores, the risk group that the patient belongs to could be obtained. *SDRLN* short diameter of residual lymph node, *VEGF* vascular endothelial growth factor, *GPS* Glasgow prognostic score.

**Figure 8:**
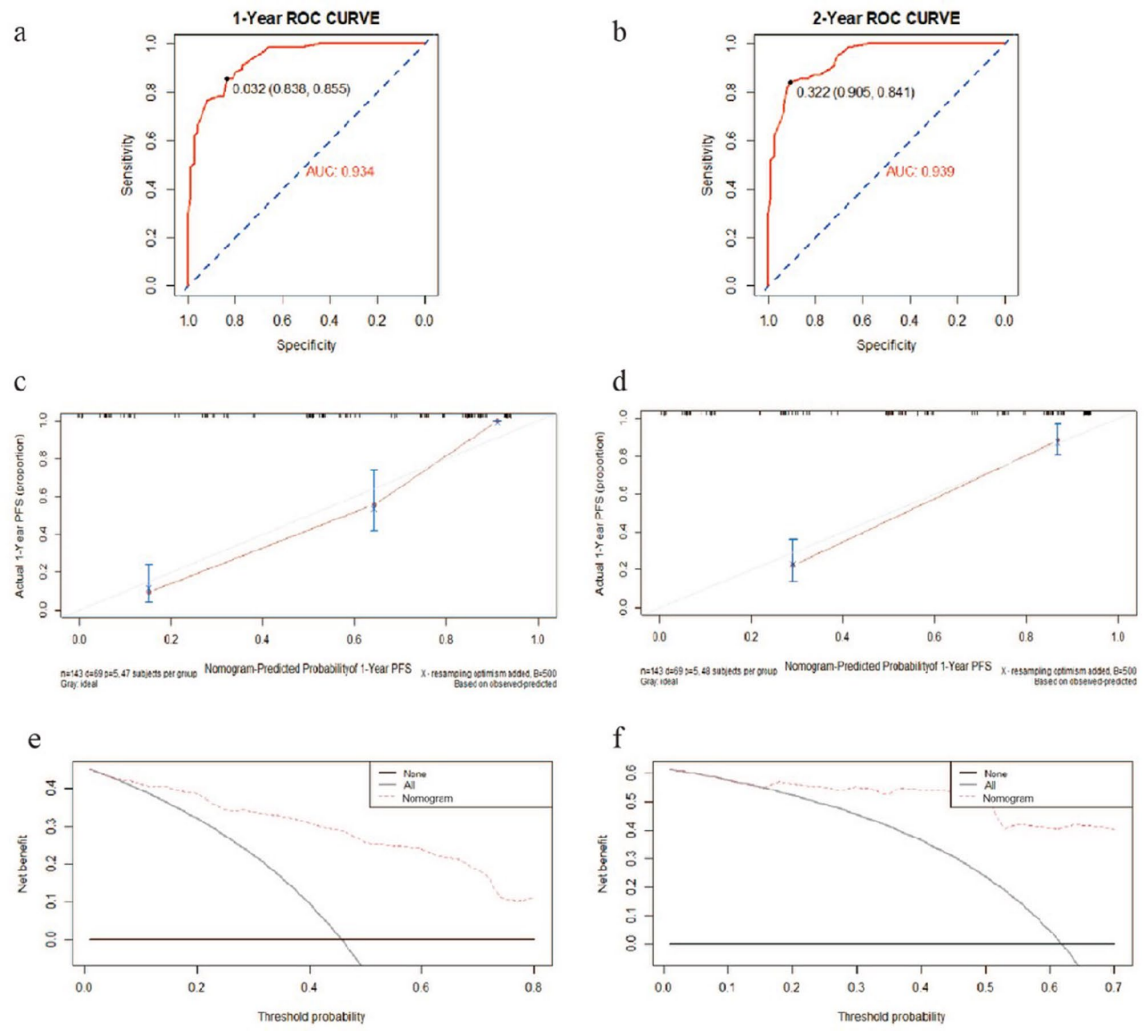
The ROC curves, calibration curves and DCA of the nomogram. (**a**, **b**) The ROC curves to predict 1-, and 2-year PFS, and the AUCs were 0.934 (threshold = 0.032, Specificity = 83.8%, Sensitivity = 85.5%) and 0.939 (threshold = 0.322, Specificity = 90.5%, Sensitivity = 84.1%), respectively. (**c**, **d**) Calibration curves showing the probability of 1-, and 2-Year PFS between the nomogram prediction and the actual observation, respectively. Perfect prediction would correspond to a slope of 1 (diagonal 45-degree gray line). (**e**, **f**) DCA of the nomogram predicting the probability of 1-, and 2-Year PFS respectively. The x-axis represents the threshold probabilities, and the y-axis measures the net benefit calculated by adding the true positives and subtracting the false positives. The horizontal line along the x-axis assumes that progression-free survival occurred in no patients, whereas the solid gray line assumes that all patients will have progression-free survival at a specific threshold probability. The red dashed line represents the net benefit of using the nomogram.

Combined the results described above, we integrated several independent risk factors to establish the prognostic nomogram (Fig. 7), involving differentiation, TNM stage, SDRLN, the expression of VEGF and GPS 3 months after radiotherapy. The C-index (value = 0.836) and ROC curves (Fig. 8a,b) were used to evaluate the discrimination power of the nomogram in prognostic prediction. The AUC of 1-, and 2-year PFS prediction probability was 0.934 (threshold = 0.032, specificity = 83.8%, sensitivity = 85.5%) and 0.939 (threshold = 0.322, specificity = 90.5%, sensitivity = 84.1%), respectively. In addition, calibration curves for the nomogram was coincident with the reference line (Fig. 8c,d), which indicated a high degree of credibility. The DCA curves used to inform clinical decisions were presented in Fig. 8e,f.

## Discussion

In our study, we conformed the important value of combined detection of peripheral blood VEGF end of radiotherapy and CLR end of radiotherapy with respect to clinical response evaluating, as well as combined detection of peripheral blood VEGF 3 months after radiotherapy and GPS 3 months after radiotherapy in progression-free survival prediction. A prognostic prediction model was established to provide an effectively prognosis prediction method, in a purpose to guide clinical treatment in the future.

VEGF, as the strongest factor inducing of angiogenesis^19^, can directly act on vascular endothelial cells, release proteases and degrade the extracellular matrix, thus promoting the growth of new blood vessels^20^. It can also be secreted to the periphery and be detected in peripheral blood. It was considered as a broad-spectrum hematological tumor biomarker as early as 1994^21^. Previous researches of VEGF in a variety of cancers^22–24^, including esophageal cancer, kidney cancer, colorectal cancer, etc., have confirmed its correlation to the treatment response and prognosis of tumor. Previous in vivo and in vitro studies have demonstrated that VEGF may be a potential radiosensitivity indicator for prognosis in ESCC^22,25^. In the study of Yen-Hao Chen^26^, the expression of VEGF can be used as an independent therapeutic response factor for esophageal cancer patients underwent radiotherapy or chemotherapy. Our logistic regression analysis more convincingly identifies that patients with high expression of VEGF at the end of radiotherapy have poor clinical response. In addition, our study found the VEGF expression level before and during radiotherapy has no correlation with clinical response. As several studies proposed that the expression level of VEGF before treatment is not significantly different from that of normal people^27–29^, and the registration is high. Previous research has demonstrated that VEGF levels end of radiotherapy were significantly associated with pathological response^22^, is consistent with our study. Radiotherapy intensified hypoxia in tumor microenvironment, increased production of hypoxia inducible factor 1α (HIF-1α)^4^, resulting in increasing cancer proliferation and metastasis through sustained secretion of VEGF for patients with worse clinical response. An association of VEGF with PFS in patients with ESCC cancer has been reported^22^. This study proved that higher levels of VEGF lead to higher risks of tumor proliferation and migration, which triggered a worse prognosis. However, most studies did not group the value of VEGF^30,31^, and continuous variables are not conducive to the clinical decision treatment. Our study compares the AUCs value and obtains the optimum cutoff value with the highest specificity and sensitivity, which can guide clinical application.

The inflammation biomarkers are associated with aggressive tumor characteristics in various tumors^32,33^. CLR, as a promising new marker for predicting surgical and oncological outcomes in colorectal cancer^14,34^, can reflect systemic inflammatory response and immune response at the same time. Okugawa et al.^14^ proposed that CLR can be used as an effective marker for perioperative and postoperative management of patients with colorectal cancer. A study of esophageal cancer showed that a high preoperative CLR was significantly associated with clinicopathological factors for disease development and CLR can be a more reliable biomarker of a poor outcome than other combinations of inflammation biomarkers^35^. Although our research believes that CLR is not a predictor of PFS, but the level of CLR at the end of radiotherapy is an independent predictor of the clinical response of ESCC patients, which further expands the scope of CLR application. It may be due to the different research objects we included. Therefore, a further validation study will be needed to confirm the value of CLR of ESCC. GPS is an inflammation biomarker based on CRP and ALB, which has been shown highly discussed in gastrointestinal tumors including ESCC before^36–38^. A meta-analysis showed that elevated GPS in patients with esophageal cancer is related to more aggressive tumor biology and poor PFS or OS^37^. Another study on esophageal cancer patients undergoing curative esophagectomy demonstrated that high expression of GPS is significantly associated with poor survival and tumor recurrence^39^. As shown in our study, the expression of GPS 3 months after radiotherapy can indeed be used as an independent predictor of PFS prediction probability, further confirming this view. A possible underlying mechanism that may explain the high GPS levels of patients with radiotherapy concerns inflammation or nutritional status, which is closely related to the signaling pathways of CD64/PI3k/Akt and MAPK/ERK signaling pathways induced by CRP and, stimulate tumor growth and worsen disease progression^16,40^. CAR is an improved inflammation biomarker based on GPS. Studies in esophageal cancer, lung cancer, liver cancer and other cancers show that CAR is more closely related to prognosis than CRP^12,41,42^, NLR (Neutrophil to Lymphocyte Ratio), PLR (Platelet to Lymphocyte Ratio) or other common inflammation biomarkers. Our results showed that CAR as the optimized biomarker in esophageal cancer cannot be used as an independent predictor of the prognosis of esophageal cancer. On the contrary, the classic GPS can be used to evaluate the prognosis, same as the study of Liu^43^. They believe that if a patient receives chemoradiotherapy, CAR has no correlation with the prognosis. Therefore, this improved indicator needs further research in predicting the prognostic survival of cancer patients.

For the first time, we found that combined detection method of peripheral blood VEGF and inflammation biomarkers levels with larger AUC can achieve better diagnostic performance, whether in terms of clinical response or prognostic prediction. This may be explained by the integration of inflammation, immunity and nutrition^44^. The combined detection of VEGF and CLR levels at the end of radiotherapy can evaluate clinical response, which is believed to be related to the inhibition of tumor by acute inflammation caused by radiotherapy and oxidative stress^20^. Increased expression of VEGF during radiotherapy indicates upregulation of tumor tissue perfusion, which can induce systemic inflammation, accompanied by a decrease in hypoxia^4^, indicating poor sensitivity to radiotherapy and poor clinical response^45^. Additionally, VEGF is not only activated and released by various cells of the inflammation and immune system at the inflammation site to induce angiogenesis, but also directly acts as a part of the positive feedback loop to active immune cells^6^. It requires certain time for feedback so that combined detection at 3 months after radiotherapy in our research can predict the prognosis by reflecting the host immune reserve capacity^40^. Given the above evidence, combined detection can be a more reliable prognostic method for response and prognosis.

The nomogram prediction model has been widely used in breast cancer, colorectal cancer, liver cancer and other common solid tumors^46–48^, but there is limited research on esophageal cancer. According to a recent study^49^, a nomogram was constructed to predict the survival of patients with metastatic esophageal cancer extracted from the Surveillance, Epidemiology, and End Results database, showing the model can minimize the variability of patient data collection and improve the general applicability of the research results, as well as our model. Their model only incorporates clinical baseline characteristics with the c-index value of 0.762. The c-indexes of most of the other esophageal cancer studies were range from 0.65 to 0.85 (average = 0.075)^50^. The high c-index of our model (c-index value = 0.836) shows that it has a high distinguishing ability with the combined detection of VEGF and GPS. The AUCs of the ROC curve for the prognostic model for predicting the 1-, and 2-year PFS indicating that the model has a good performance for prognosis prediction. Formulating treatment strategies based on our nomogram can have high net benefits according to the DCA curves. VEGF has been a well-established therapeutic target, approved for the clinical treatment of esophageal cancer^51^. Evidence from clinical and preclinical studies indicates that anti-inflammatory therapy can suppress inflammation and immune response^15,33^, thus improving prognosis. Considering these findings, our nomogram model could identify high-risk population with poor prognosis, allowing timely and targeted specific therapies, providing novel therapeutic strategies for the treatment of ESCC, which has a good application prospect.

In addition, we also found that the SDRLN after radiotherapy can be used as an independent factor for evaluating treatment response or prognosis. It confirms the importance of short diameter of the lymph nodes in esophageal cancer^52^. Although the lymph node is considered to be an important factor in the prognosis of tumors, there are very few studies on measuring the short diameter of the lymph nodes in tumors. In the RECIST criteria^18^, only lymph nodes with a short diameter greater than or equal to 15 mm are defined as metastatic new lymph nodes. However, the short diameter of metastatic lymph nodes, especially those in the upper mediastinum, are usually less than 15 mm. It may lead to insufficient assessment of disease progression and prognosis. Data-based measurement of the short diameter of lymph nodes can make up for this shortcoming instead of calculating volume indicators by the previous use of barium meal, CT combined with MRI et al. method^53^. It provides additional value for precise predictions of patient clinical response and prognosis, and is more economical, simple, and faster.

As far as we know, this study is the first systematic and dynamic study to integrate angiogenesis and inflammation biomarkers in patients with ESCC. But we admit that there are still some shortcomings, namely, single-center research, a small research sample size, and the lack of external verification of the model. We look forward to multi-center, large sample, forward-looking collaborative research in the future to further prove the conclusions of this research.

In conclusion, combined detection of peripheral blood VEGF and inflammation biomarkers have prognostic value for the clinical response assessment and prognostic prediction. The nomogram based on basic clinical data, VEGF and GPS could be used as an accurately prognostic prediction for patients with non-operative ESCC.

## Supplementary Information

Supplementary Figures.

## Data Availability

The data generated or analyzed during this study are available from the corresponding author upon reasonable request.
