# Supplementary material for: Combined detection of peripheral blood VEGF and inflammation biomarkers to evaluate the clinical response and prognostic prediction of non-operative ESCC
Source: Sci Rep. 2021 Jul 27;11:15305. doi: 10.1038/s41598-021-94329-8 (PMC8316563; doi:10.1038/s41598-021-94329-8)
Supplement: Supplementary file 1 — Supplementary Figures. [file 41598_2021_94329_MOESM1_ESM.pdf]

Title:

Combined Detection of Peripheral Blood VEGF and Inflammation Biomarkers to Evaluate the Clinical Response and Prognostic Prediction of Non-operative ESCC

Author:

Yuanyuan Ma
782640291@qq.com
The Affiliated Huai'an Hospital of Xuzhou Medical University, The Second People's Hospital of Huai'an CN

Xinyu Su
15261798461@163.com
The Affiliated Huai'an Hospital of Xuzhou Medical University, The Second People's Hospital of Huai'an CN

Xin Li
958858419@qq.com
The Affiliated Huai'an Hospital of Xuzhou Medical University, The Second People's Hospital of Huai'an CN

Xiaohui Zhi
zhi1490@126.com
First Affiliated Hospital of Nanjing Medical University CN

Kan Jiang
jiangkan@zju.edu.cn
First Affiliated Hospital Zhejiang University CN

Jianhong Xia
xjh_ha@163.com
The Affiliated Huai'an Hospital of Xuzhou Medical University, The Second People's Hospital of Huai'an CN

Hongliang Li
2750238955@qq.com
The Affiliated Huai'an Hospital of Xuzhou Medical University, The Second People's Hospital of Huai'an CN

Chen Yan
865345704@qq.com
The Affiliated Huai'an Hospital of Xuzhou Medical University, The Second People's Hospital of Huai'an CN

Corresponding author:

Liqing Zhou
zlq-hill@163.com
The Affiliated Huai'an Hospital of Xuzhou Medical University, The Second People's Hospital of Huai'an CN

Supplementary Figures:

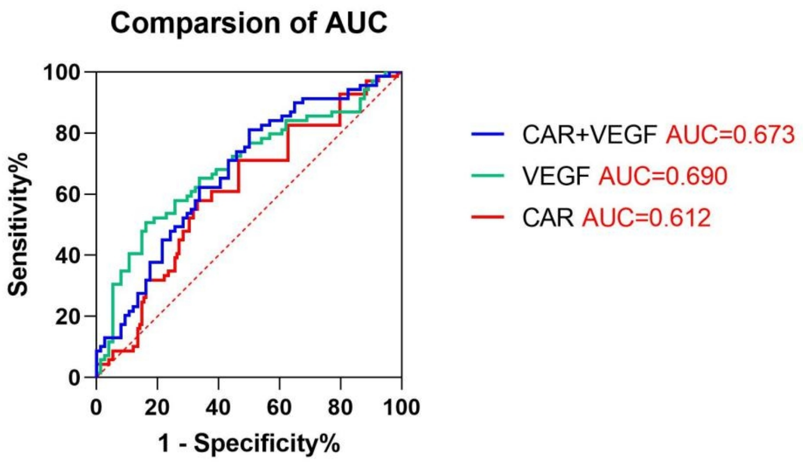

Supplementary Figure S1. Comparison of the AUCs for the clinical response assessment of combined detection of VEGF and CAR (AUC=0.667, p<0.0001), VEGF (AUC=0.617, p=0.003), CAR (AUC=0.622, p<0.0001) before radiotherapy for ESCC patients.

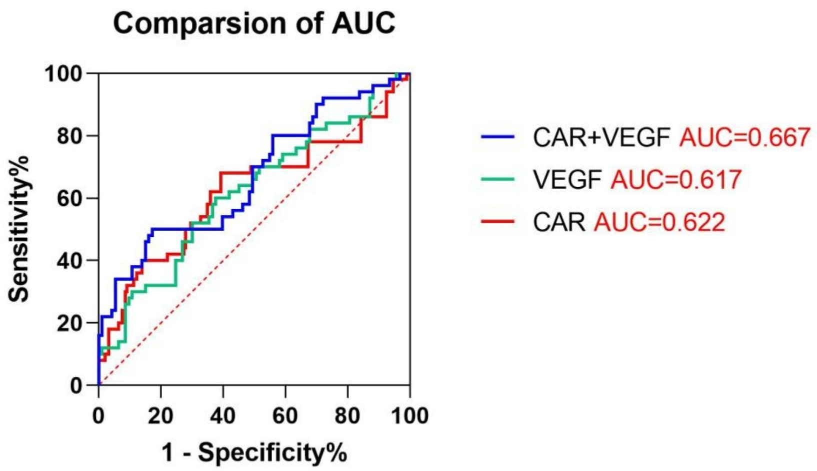

Supplementary Figure S2. Comparison of the AUCs for prognostic prediction of combined detection of VEGF and CAR (AUC=0.673, p<0.0001), VEGF (AUC=0.690, p=0.003), CAR (AUC= 0.612, p<0.0001) before radiotherapy for ESCC patients.
